# The Role of the Periosteum in Bone Formation From Adolescence to Old Age

**DOI:** 10.7759/cureus.76774

**Published:** 2025-01-01

**Authors:** Panagiotis Panagakis, Konstantinos Zygogiannis, Ilias Fanourgiakis, Dimitrios Kalatzis, Konstantinos Stathopoulos

**Affiliations:** 1 3rd Orthopedic Department, KAT General Hospital, Athens, GRC; 2 Scoliosis and Spine Department, KAT General Hospital, Athens, GRC; 3 Orthopedics and Traumatology, Laiko General Hospital of Athens, Athens, GRC; 4 Laboratory for Research of the Musculoskeletal System, National and Kapodistrian University of Athens, Athens, GRC

**Keywords:** age and ageing, bone biology, bone formation, osteogenesis, periosteum

## Abstract

Bone formation is a complex process involving the coordinated activity of many different cell types, including osteoblasts and osteocytes. The periosteum is a dense membrane of connective tissue that covers the outer surface of bones and is essential for the growth, repair, and maintenance of osseous tissue. The present study aims to summarize the contribution of the periosteum in bone formation from adolescence to adulthood and old age. This is a narrative literature review using the PubMed electronic internet database. The search was based on the keyword "periosteal bone formation". Inclusion criteria were preclinical or clinical studies evaluating the role of the periosteum in bone formation. Non-English studies were excluded. The original search provided 126 published papers. After inclusion and exclusion criteria, we finally accepted 20 articles for our current review. After checking the references list of the included studies, 14 more studies were added, leaving 34 studies for the present review. Across the lifespan, periosteal bone formation undergoes dynamic changes. During adolescence, the periosteum is highly osteogenic and actively contributes to rapid bone growth. In adulthood, it plays a role in maintaining bone strength and adapting to mechanical loading. In adulthood, the periosteum continues to provide a source of osteoprogenitor cells, which contribute to the ongoing process of bone remodeling and repair. At more advanced ages, the response of the periosteum to hormones and cytokines in terms of bone formation decreases; however, the power of osteogenetic differentiation of periosteal cells may be preserved.

## Introduction and background

The periosteum is a dense connective tissue membrane that covers the outer surface of bones, except at the joints where it is replaced by articular cartilage [1-2]. The periosteum consists of two layers. The outer fibrous layer is composed of dense irregular connective tissue, primarily made up of collagen fibers, which provide strength and protection to the underlying bone [1-2]. Blood vessels, nerves, and lymphatic vessels supplying the bone pass through this layer. The inner layer of the periosteum is the cambium or osteogenic layer. It is more cellular and contains osteogenic cells (osteoblasts and osteoprogenitor cells) that are responsible for bone growth and repair [1-2]. The cambium layer is essential for bone formation during bone growth and repair processes. Fibrous bundles called Sharpey's fibers extend from the fibrous layer into the bone matrix. These fibers anchor the periosteum firmly to the bone, providing additional strength and stability [2].

The periosteum serves several important functions that contribute to the overall health, maintenance, and functionality of bones. The periosteum contains osteogenic cells, including osteoblasts and osteoprogenitor cells, which are crucial for bone formation and growth, contributing to appositional growth. The periosteum is highly vascularized, with blood vessels that penetrate the tissue to supply oxygen, nutrients, and minerals to the underlying bone [3]. The periosteum, due to its extensive vascular network, has been characterized as the "umbilical cord of bone," providing 70-80% of the blood supply to the cortical part of the bone [4]. The fibrous outer layer of the periosteum provides a protective covering for bones, helping shield them from external forces and potential injuries. Moreover, the periosteum serves as a point of attachment for muscles and tendons. Sharpey's fibers, which are collagen bundles extending from the periosteum into the bone, anchor muscles and tendons to the bone surface, providing stability during movement. The periosteum contains sensory nerves that convey pain and proprioceptive sensations.

In response to bone injury or fractures, the periosteum is involved in the initial stages of the healing process. Osteoprogenitor cells in the cambium layer can differentiate into osteoblasts, contributing to the formation of callus and new bone tissue during the repair process [5]. It also plays an important role in the bone remodeling process in which the old bone tissue is degraded and replaced with new bone tissue [6]. Increased mechanical stress stimulates the periosteum to initiate bone remodeling, a process that strengthens bones in response to the demands placed upon them. Furthermore, during embryonic development, the periosteum contributes to hematopoiesis (blood cell formation) before the bone marrow becomes the primary site for this process [2]. In addition to its main functions, periosteum plays a role in the pathogenesis of bone metastases. Cancer cells migrating to bone stimulate the periosteum, leading to osteoblast activation and bone formation, triggering the formation of bone metastases [7-8].

Bone formation during adolescence is a dynamic and critical process that significantly influences an individual's skeletal health and development. During adolescence, pubertal growth spurt occurs, a process characterized by rapid skeletal growth, changes in bone mineral density, and the attainment of peak bone mass [9]. It is characterized by a sudden increase in height and weight, as well as changes in body composition. This growth spurt typically occurs between the ages of 10 and 16 in boys and between the ages of 8 and 14 in girls, although the timing can vary widely among individuals [10]. While both boys and girls experience pubertal growth spurts, there are some differences in the timing, duration, and magnitude of growth between the two sexes. Boys typically experience their pubertal growth spurt later than girls. On average, boys begin puberty around the ages of 10 to 12, with the peak growth spurt occurring around age 14. Girls, on the other hand, usually enter puberty earlier, with the initial stages beginning around ages 8 to 10 and the peak growth spurt typically occurring around age 12. The pubertal growth spurt tends to be longer in girls than in boys. Girls may continue to grow steadily for about two to three years after the onset of puberty while boys may experience a shorter period of rapid growth, usually lasting around two years [11]. Boys generally experience a greater increase in height during their pubertal growth spurt as compared to girls. This is due to the influence of testosterone, which promotes the growth of long bones and muscle mass in boys. On average, boys can gain several inches in height during their growth spurt, whereas girls typically experience a more gradual increase in height [12]. Along with the pubertal growth spurt, both boys and girls develop secondary sexual characteristics, but the timing and nature of these changes differ between the sexes. Girls typically experience breast development, widening of the hips, and the onset of menstruation during puberty while boys may experience the growth of facial hair, deepening of the voice, and increased muscle mass [13].

Bone formation during adolescence is orchestrated by the interplay of hormones, osteoblast activity, mineralization, mechanical loading, and nutritional factors [14]. During adolescence, growth plates are highly active, and longitudinal bone growth occurs as new bone is formed at the epiphyseal plates. The proliferation and differentiation of chondrocytes in the growth plates contribute to the lengthening of bones. Osteoblast activity is heightened, especially on the periosteal surface of bones, leading to appositional growth, thus increasing bone width. Bone mineral density increases during adolescence, reaching its peak in early adulthood. Areal bone density increases with growth during childhood, is highest around 20 years of age, and declines thereafter. The increase in volumetric bone mineral density during adolescence is primarily attributed to bone growth and mineralization [15]. Peak bone mass is a critical factor in determining bone health later in life. The greater the peak bone mass, the lower the risk of osteoporosis and fractures later in life [16]. Adequate nutrition, particularly sufficient intake of calcium, vitamin D, and other minerals, is crucial for bone mineralization and optimal bone formation. Adolescents experiencing growth spurts have increased nutritional requirements to support the demands of bone growth [17]. Growth hormone (GH) and insulin-like growth factor-1 (IGF-1) are key stimulators of bone growth. They promote the proliferation of chondrocytes in the growth plates and the activity of osteoblasts. Estrogen and testosterone also influence bone growth and maturation. They contribute to the closure of the epiphyseal plates and play a role in achieving peak bone mass, marking the end of longitudinal bone growth [14].

As a person reaches adulthood, the rate of bone formation begins to decelerate. Bones have reached maximum strength and density by age 30 [18]. However, bone formation continues throughout adulthood, albeit at a slower rate. In this stage, the process of bone remodeling dominates. Osteoclasts degrade old or damaged bone tissue, and osteoblasts replace it with new bone. This continuous remodeling maintains bone strength, repairs micro-damage, and adapts bone structure to changing mechanical demands. During adulthood, bones become wider as part of the natural process of skeletal growth and development. The impact of genetic factors becomes less important as a person reaches their maximum bone mass. Diet and hormonal factors also remain important in adulthood, as inadequate intake of key nutrients or hormonal imbalances can lead to impaired bone formation. Strategies to prevent excessive bone loss include maintaining a healthy lifestyle, adequate nutrition, and regular weight-bearing exercise. Conversely, a sedentary lifestyle can lead to bone loss and an increased risk of osteoporosis [19-20].

At older ages, there is a natural decline in bone density and changes in bone architecture. Osteoblastic activity may decrease, leading to a gradual imbalance between bone formation and resorption. This age-related decline in bone mass is more pronounced in women after menopause due to decreased estrogen levels. This disease is known as osteoporosis and can increase the risk of fractures [21]. Hormonal changes associated with aging, such as decreased estrogen levels in postmenopausal women and declining levels of growth hormone, affect the balance between bone resorption and formation, contributing to bone loss. Women are at a higher risk of osteoporosis than men, mainly due to the drop in estrogen levels that occurs after menopause. Inadequate protein intake can lead to loss of muscle mass, which can contribute to reduced bone strength. Adequate nutrition, particularly calcium and vitamin D, remains important in old age to support bone formation. Weight-bearing exercises, even in the elderly, can help maintain bone density and improve balance, reducing the risk of falls and fractures. Smoking and alcohol consumption and long-term use of medications such as glucocorticoids are also modifiable factors, which can negatively affect bone formation in old age [22-23].

The present study aims to summarize the contribution of periosteum in bone formation from adolescence to adulthood and old age.

## Review

Materials and methods

This narrative literature review was conducted using the PubMed electronic database. The search utilized the keyword “periosteal bone formation”. Inclusion criteria included preclinical and clinical studies that investigated the biological, structural, or functional role of the periosteum in bone formation or remodeling across different life stages, from adolescence to old age. Studies on human subjects or animal models with translational relevance, as well as original research, systematic reviews, meta-analyses, and clinical reviews, were considered. Articles discussing periosteum-related mechanisms, such as fracture healing, age-related changes, or osteoporosis, were prioritized. Since our study emphasizes the biological process and mechanisms, we did not conduct a statistical analysis. Only publications in English were included, and studies published in non-English languages were excluded.

Results

The original search provided 126 published papers. After applying the inclusion and exclusion criteria, we finally accepted 20 articles for our current review. After checking the references list of the included studies, 14 more studies were added, leaving 34 studies for the present review [24-57]. Additional criteria were not applied after the initial inclusion/exclusion. The studies included in our review span the period from 1988 to 2023.

The role of periosteum in bone formation in adolescence

The osteogenic potential of periosteum is essential for bone formation, bone remodeling, and fracture repair during adolescence, playing a key role in the recruitment and differentiation of bone cells. Periosteum contains an osteogenic (cambium) layer that harbors osteogenic cells, including osteoblasts, osteoclasts, osteoprogenitor cells, and mesenchymal stem cells [27]. Osteoblasts are responsible for synthesizing new bone matrix, and osteoprogenitor cells can differentiate into osteoblasts. Through osteoblasts-derived bone formation, the periosteum participates in this remodeling process, contributing to the maintenance and adaptation of bone structure.

In cases of fractures during adolescence, the periosteum plays a crucial role in the initial stages of the healing process. Osteoprogenitor cells in the cambium layer can differentiate into osteoblasts, contributing to the formation of callus and new bone tissue. Moreover, periosteal cells activate the expression of the vascular endothelial growth factor (VEGF), which favors revascularization and bone repair after fractures. The participation of the periosteum in bone regeneration begins immediately after the trauma, within the first 24-48 hours when a phase of acute inflammation is observed in the periosteum [48]. Periosteal cells begin to multiply and the periosteum itself increases in thickness. This process has been described as a periosteal reaction [46]. In a study by Colnot, it was shown that the pluripotent mesenchymal cells of the cambium layer of the periosteum are responsible for the creation of the bone callus. By extension, osteoprogenitor cells actively participate in osteogenesis, chondrogenesis, and angiogenesis, ultimately leading to the vascularization of the neoplastic tissue and ultimately bone remodeling [46].

Puberty is characterized by rapid bone growth, and the periosteum mainly contributes to appositional growth. The osteoprogenitor cells of its cambium layer are activated through insulin-like growth factors 1 (IGF-1) and bone morphogenetic proteins (BMPs) and turn into osteoblasts [25,58]. Periosteal osteoblasts add new bone tissue to the outer surface of existing bones, increasing bone diameter and providing structural support. The produced osseous tissue is deposited around and between the periosteal vessels resulting in the formation of periosteal striae, which, in later stages, coalesce around the periosteal vessels, thus producing Haversian canal osteons surrounded by concentric rings of bone matrix, forming cortical bone [2]. Periostin, also known as osteoblast-specific factor 2 (OSF-2), is a protein mainly located in preosteoblasts and secreted into the extracellular matrix of the periosteum cambium layer. During childhood and adolescence, periostin contributes to the expansion of the periosteum by facilitating osteoblast differentiation and mineralization of the bone matrix [28]. Recent experimental studies showed that the periosteum also participates in the longitudinal bone growth. PTH-related Peptide (PTHrP) produced in the outer fibrous layer of the periosteum regulates bone formation at the diaphyseal-metaphyseal junction of long bones [57].

In children and adolescents, the periosteum is highly vascularized, ensuring an abundant blood supply to the underlying bone tissue. This vascular network delivers nutrients, oxygen, and signaling molecules crucial for the metabolic activities of bone-forming cells, facilitating intracellular communication. Moreover, periosteal cells activate vascular endothelial growth factor (VEGF) expression enhancing revascularization after fractures [48]. Due to its high vascularity, the periosteum contains an abundance of endothelial pericytes that are in physical contact with capillary endothelial cells, with the ability to differentiate into osteoblasts. Cultured pericytes produce alkaline phosphatase (ALP), as well as non-collagenous proteins of bone matrix, contributing to bone formation [1].

The role of the periosteum in bone formation during puberty is also influenced by various hormones, such as growth hormone (GH), estrogen, and testosterone. GH is produced by the pituitary gland and stimulates the production of the growth factor IGF-1, which plays a critical role in bone formation [56]. In osteopenic rats, GH has been found to mitigate the loss of periosteal bone formation rate [31]. Periosteal cells express both estrogen and androgen receptors, as well as enzymes vital for the metabolism of sex steroids [45,55,59]. Estrogen promotes bone formation by stimulating the activity of osteoblasts while testosterone promotes bone formation by increasing the activity of osteoprogenitor cells. During puberty in males, the periosteum expands under the influence of androgens with little change in medullary diameter so that cortical thickness increases. In females, periosteal expansion stops and medullary diameter decreases as medullary bone formation takes place [44]. In females, both periosteal apposition and endocortical resorption occur. In males, endocortical resorption is minor [60].

Mechanical loading of bone during adolescence also plays a critical role in bone formation, and the periosteum is a key mediator of this process. Mechanical loading refers to the pressure and stress placed on bones during physical activity, which stimulates osteoblasts to produce new tissue. Bones have the ability to adapt to the mechanical loads presented during physical activities, changing their geometry, strength, and mass [61]. During exercise, the mechanical loads exerted on the bones by the tendons of the muscles are relatively greater than the forces exerted on the bones by gravity [54]. According to Frost's Mechanostat theory, "the application of load to the bone increases its density and improves its microarchitecture and strength, whereas, on the contrary, the absence of applied load causes osteoporosis and a decrease in its strength" [62]. This phenomenon is particularly evident during periods of skeletal growth such as adolescence. According to this theory, physically active children have stronger bones than their peers with less physical activity [61]. The degree of bone response to physical activity is extremely important in achieving maximal bone mass. The periosteum plays a role in this process as, through the nerve fibers it contains, it detects mechanical loading and transmits signals to the osteoprogenitor cells of its inner layer, stimulating osteoblastogenesis and bone formation. Weight-bearing activities and mechanical loading during adolescence stimulate the periosteum, leading to increased bone formation and remodeling [63]. Studies have shown that people who participate in regular physical activity during adolescence have higher bone mineral density and a lower risk of osteoporosis later in life [64-65].

The role of the periosteum in bone formation during adolescence can also be influenced by various environmental factors, such as diet and physical activity. Adequate intake of calcium, vitamin D, and other nutrients is essential for the metabolic activities of osteoblasts and the synthesis of bone matrix [66].

The role of periosteum in bone formation in adulthood

The inner cambium layer of the periosteum reaches its maximum thickness in the fetus and becomes progressively thinner with age [49]. In adults, the thickness of the cambium layer is significantly smaller so that it cannot be easily distinguished from the outer fibrous layer. The density of blood vessels and the number of periosteal fibroblasts also decrease with age, so that in the adult, the periosteum is only apparent as a very thin layer of tissue surrounding bony structures [67]. However, it retains its ability to increase when activated by mechanical loading or fracture repair [1].

The process of bone formation in adulthood is known as bone formation by periosteal apposition. This process occurs in the cambium layer of the periosteum and involves the activation of osteoprogenitor cells which differentiate into osteoblasts. Osteoblasts then migrate to the bone surface and begin to secrete collagen and other non-collagenous proteins that form the bone extracellular matrix. Osteoblasts also produce enzymes necessary for the mineralization of bone matrix. As new osseous tissue forms, osteoblasts become trapped within the bone matrix and differentiate into osteocytes.

The process of periosteal apposition is regulated by a complex interplay of factors, including the effect of mechanical load, hormones, growth factors, and cytokines. Mechanical stress is the most powerful stimulator of periosteal apposition. Bones subjected to repetitive mechanical stress, such as those in the lower extremities, show increased bone formation in response to the mechanical stimulus [43,50,53]. Experimental studies have shown that CD68+ F4/80+ monocytes located in the cambium layer of the periosteum respond to mechanical stimuli by producing the transforming growth factor-β (TGF-β), which stimulates bone formation [26]. The secreted frizzled-related protein 4 (SFRP4), a Wnt signaling antagonist, is expressed by periosteal cells and plays a central role in their differentiation and function [24]. Dickkopf-1 (DKK1), another Wnt signaling antagonist, regulates periosteal bone formation in inflammatory arthritis [37]. Osteoblastin Wnt1 regulates periosteal bone formation in adult mice [41]. The canonical Wnt signaling regulates mechanical stress-induced periosteal none formation [42]. Moon et al. observed that extracellular proteins secreted by mature osteoblasts and osteocytes suppress the proliferation of periosteal osteoprogenitors by blocking Wnt signaling in a paracrine manner [35].

The most important hormone in periosteal apposition is parathyroid hormone (PTH), which stimulates bone resorption and the release of calcium into the circulation [52]. Increased levels of calcium in the bloodstream then stimulate bone formation and periosteal apposition [52]. During menopause, reduced serum estrogen levels are associated with periosteal expansion [68]. Growth factors, such as IGF-1, FGF, and BMPs, also play a critical role in regulating periosteal apposition, by stimulating the differentiation of osteoprogenitor cells into osteoblasts and promoting bone formation [38-40,69]. Cytokines such as TNF-a and IL-1 can inhibit periosteal apposition by promoting bone resorption and decreasing bone formation [70]. On the other hand, IL-6 signaling in osteoclasts promotes periosteal bone formation [33]. IL-17A deficiency has been found to promote periosteal bone formation in a model of inflammatory arthritis [36]. In animal studies, overexpression of hedgehog peptides may restore periosteal bone formation [32]. Conditional disruption of the miR17-92 cluster in collagen type I-producing osteoblasts results in reduced periosteal bone formation [34].

Throughout adulthood, bones undergo continuous remodeling, involving the simultaneous processes of bone resorption and bone formation. The periosteum contributes to this remodeling process by providing a source of osteoblasts, which are responsible for forming new bone tissue. Periosteal apposition is a critical process in adulthood because it allows bones to adapt to changes in mechanical stress and maintain their strength and integrity, protecting against fracture. At the same time, it plays an important role in the restoration of bones after fractures. Periostin mediates cortical bone response to mechanical forces [30]. Periostin expression is increased four-fold within three days after fracture [51]. Osteoprogenitor cells in the cambium layer can differentiate into osteoblasts, contributing to the formation of callus and new bone tissue at the site of injury. Periosteal PTHrP regulates cortical bone remodeling during fracture healing [71]. However, the extent to which the process of periosteal apposition occurs in adulthood after closure of the epiphyseal plates is controversial and the results of related investigations are heterogeneous. Periosteal apposition continues to occur during adulthood as part of the dynamic process of bone remodeling, adaptation, and repair in response to mechanical stress, bone loss, and fracture healing [67].

The role of periosteum in bone formation in old age

Along with the structural changes of the periosteum with increasing age, there is also a decrease in the osteogenic potential of the tissue, which can positively influence osteogenesis under appropriate stimuli, but to a lesser extent as age increases [72]. These changes include a decrease in the number of osteoprogenitor cells and a decrease in the production of growth factors and cytokines. The number of osteoprogenitor cells within the cambium layer of the periosteum decreases with age, reducing the pool of osteoprogenitor cells available to differentiate into osteoblasts [28]. In addition, osteoprogenitor cells that remain in the periosteum become less responsive to stimuli that promote differentiation into osteoblasts, such as mechanical loading. The production of growth factors (IGF, BMPs) and cytokines (TNF-a, IL-1) within the periosteum also decreases with age. Reduced production of these factors can impair the ability of the periosteum to form new bone and contribute to the development of osteoporosis [73].

Although old age does not seem to inhibit the regenerative properties of the periosteum, some age-related changes include a decrease in the number of periosteal fibroblasts, thickness of the fibrous layer, number of osteoblasts, collagen production, and vascular density [47,74]. Experimental studies have shown a limited number of osteoclasts in younger animals. In contrast, in aged animals, the number of periosteal osteoclasts is increased [49]. In the periosteum of young rats, both layers are highly vascularized, whereas in mature rats, blood vessels are mainly found in the fibrous layer. The higher degree of vascularity in the periosteum of young rats suggests a role in providing nutrients and osteoprogenitor cells. Both the thickness and the number of cells in the periosteum of the diaphysis decreased with age [75]. Interestingly, periosteal cells in aged humans retain a high capacity for proliferation and differentiation, although their ability to differentiate into chondrogenic and lipogenic lineages declines with age [76].

Hormonal changes associated with aging, such as declining levels of estrogen and testosterone, can influence bone metabolism. Changes in hormonal signaling may affect the responsiveness of the periosteum to hormonal cues that regulate bone formation. The reduced hormone levels seen in old age limit the potential for periosteal apposition. Overall, the responsiveness of the periosteum to hormones and cytokines in terms of bone formation declines with age, although the osteogenic differentiation capacity of periosteal cells may be preserved [49].

Old age is characterized by an increased rate of osteoporosis and the use of antiosteoporotic drugs. Daily administration of PTH increases cortical bone width through preferential modeling on both periosteal and endosteal surfaces. In postmenopausal women, long-term treatment with PTH increases vertebrae and radius cross-sectional area and cortical wall thickness along with femoral neck cortical bone volume, through endocortical and periosteal surface formation. Primary hyperparathyroidism has been associated with a two- to three-fold higher periosteal bone formation rate [77]. Long-term administration with PTH in humans stimulates periosteal surfaces [78]. Bisphosphonate administration results in an increased rate of periosteal apposition, which is transient and takes place only during treatment [79]. In knockout mice, the suppression of PTH activity in osteocytes further exacerbated the loss in bone formation at the endocortical surface but inhibited bone loss at the periosteal surface [29].

## Conclusions

The periosteum is a complex structure consisting of an outer fibrous layer that provides structural integrity and an inner cambium layer that possesses osteogenetic potential, containing osteoprogenitor cells. These cells, following the effect of hormones, growth factors, and mechanical stimuli, are activated, multiply, and differentiate into osteoblasts, which produce new bone tissue. Through this mechanism, the periosteum participates in the extensive bone growth during puberty. In adulthood, bone formation in the periosteum takes place through periosteal apposition, as part of the ongoing bone remodeling that bones undergo. The response of the periosteum to hormones and cytokines, regarding bone formation, decreases with age. In old age, there is a decline in osteogenic activity, and the periosteum may become less effective in compensating for bone resorption. Understanding these changes is essential for developing strategies to support bone health and prevent age-related bone loss. Periosteum can be harvested easily in the clinical setting without much morbidity in the donor area. Thus, periosteum-derived cells are expected to be a good source of cells for bone regeneration.
